# Top-down and sideways: Herbivory and cross-ecosystem connectivity shape restoration success at the salt marsh-upland ecotone

**DOI:** 10.1371/journal.pone.0247374

**Published:** 2021-02-22

**Authors:** Kerstin Wasson, Karen E. Tanner, Andrea Woofolk, Sean McCain, Justin P. Suraci

**Affiliations:** 1 Elkhorn Slough National Estuarine Research Reserve, Royal Oaks, California, United States of America; 2 Ecology and Evolutionary Biology, University of California, Santa Cruz, California, United States of America; 3 California Department of Fish and Wildlife, Sacramento, California, United States of America; 4 Environmental Studies, University of California, Santa Cruz, California, United States of America; Bowling Green State University, UNITED STATES

## Abstract

Wetland restoration provides remarkable opportunities to understand vegetation dynamics and to inform success of future projects through rigorous restoration experiments. Salt marsh restoration typically focuses on physical factors such as sediment dynamics and elevation. Despite many demonstrations of strong top-down effects on salt marshes, the potential for consumers to affect salt marsh restoration projects has rarely been quantified. Recently, major restoration projects at the Elkhorn Slough National Estuarine Research Reserve in central California, USA provided an opportunity to examine how herbivory influences restoration success. We quantified the strength of consumer effects by comparing caged to uncaged plantings, and compared effects among plant species and sites. We used camera traps to detect which herbivores were most common and how their abundance varied spatially. Beyond characterizing consumer effects, we also tested management strategies for reducing negative effects of herbivory at the restoration sites, including caging, mowing, and acoustic playbacks of predator sounds. We found extremely strong consumer effects at sites with extensive stands of exotic forbs upland of the high marsh; uncaged restoration plants suffered heavy herbivory and high mortality, while most caged plants survived. Brush rabbits (*Sylvilagus bachmani*) were by far the most frequent consumers of these high marsh plants. Our work thus provides the first evidence of mammal consumers affecting salt marsh restoration success. Mowing of tall exotic forb cover adjacent to the marsh at one restoration site greatly reduced consumption, and nearly all monitored plantings survived at a second restoration site where construction had temporarily eliminated upland cover. Playbacks of predator sounds did not significantly affect restoration plantings, but restoration efforts in marsh communities vulnerable to terrestrial herbivory may benefit from concurrent restoration of predator communities in the upland habitats surrounding the marsh. A landscape approach is thus critical for recognizing linkages between terrestrial and marine vegetation.

## Introduction

Salt marshes comprise the dominant foundation species of temperate coastal estuaries, and provide key ecosystem services [1, 2]. Globally, anthropogenic activities have led to extensive loss of salt marshes and their associated services [3, 4]. Many restoration projects aimed at restoring lost functions and extent of salt marshes have been initiated over the past decades. While some restoration projects have been well-studied and syntheses of marsh restoration science have emerged [5–8], restoration monitoring of many projects is quite limited, and significant questions remain about factors predicting marsh restoration success or failure.

Salt marsh restoration projects typically emphasize the role of bottom-up drivers of marsh distribution. Salt marshes only occur in a very narrow band of intertidal elevation—with too much inundation they drown, but with too little they are replaced by upland plants. As such, they are vulnerable to factors that change elevation relative to water levels—for instance, subsidence from groundwater overdraft can drown marshes, and diversion of rivers that provide sediment can prevent the tracking of sea-level rise [9–12]. Most salt marsh restoration projects have thus focused on adjusting this balance between relative elevation and inundation—restoring tidal exchange to diked marshes, or adding sediment to increase marsh plain elevation [8, 13, 14]. Simply providing the correct physical conditions is often sufficient to allow for colonization of one or a few dominant species in the low-mid marsh, with colonization occurring by seeds dispersed on tides. But at the upper, landward edge of the marsh, there are often rarer species with more limited seed supply [15]. To ensure representation of this diversity and the functions these species provide, these rarer species are sometimes planted into the high marsh [16].

While physical factors are indisputably important drivers affecting the extent of salt marshes and are critical to incorporate into restoration design, biological drivers can also play a major role. In the past decades, many studies have highlighted the role that consumers—including both terrestrial and marine species—can play in shaping marsh resilience. Among marine species, snails [17], and particularly crabs [18–20], have been identified as having strong effects through consumption and/or bioturbation on some salt marsh ecosystems. Among terrestrial species, geese [21], guinea pigs [22], and domestic animals such as cows and horses [23] can have dramatic effects on salt marshes both through herbivory and trampling. Despite these demonstrations of strong top-down effects, their potential to affect salt marsh restoration projects has rarely been explored or quantified.

At the Elkhorn Slough estuary in central California, USA, about 50% of the salt marsh has been lost over the past 150 years, mostly due to diking [24]. Major restoration projects have been initiated by the Elkhorn Slough National Estuarine Research Reserve, including restoration of tidal exchange to formerly diked marshes, and raising elevation that was lost to subsidence behind dikes. Both of these restoration approaches result in bare-earth conditions; increasing the tidal range kills upland vegetation and creates a bare zone that can be colonized by returning marsh vegetation, and placement of new sediment creates extensive bare areas during construction. While the marsh dominant, pickleweed (*Salicornia pacifica*), readily disperses via tides and easily colonizes bare areas, rarer high marsh species are slow to appear. In an effort to establish these less-common species at restored sites, thousands of greenhouse-grown individuals have been planted at Elkhorn Slough Reserve restoration projects since 2017. Herbivores have the potential to affect these plantings. At Elkhorn Slough as in many salt marshes, crabs are typically limited to the more seaward portion of the marsh gradient [25] and are not likely to affect high marsh plantings. However, terrestrial herbivores may play an important role, as suggested by anecdotal observations of cropped marsh vegetation near the marsh-upland ecotone. Immediately landward of the marsh there are often large stands of tall exotic forbs [26], and rabbits are often seen using these forbs as cover. These observations motivated us to test two main hypotheses, namely 1) that herbivory by rabbits or other terrestrial species negatively affects restoration of high marsh plants, and 2) that management strategies can effectively mitigate terrestrial herbivore impacts.

We conducted four herbivory experiments at restoration sites on the Elkhorn Slough Reserve. We used cage treatments to quantify consumer effects, and compared the efficacy of individual plant cages, large cage exclosures, and removal of upland cover as strategies to protect restoration plantings. We compared herbivore impacts across different sites to assess spatial variation in herbivory and the generality of consumer effects, and tested predator calls played over loudspeakers as a method to deter herbivores. This restoration strategy was inspired by recent studies on the “ecology of fear” that have demonstrated altered consumer behavior when exposed to real or perceived predators [27, 28]. We also assessed herbivore impacts on four different marsh species to determine whether some species are more resistant to herbivores, making them suitable for restoration in herbivore-rich settings.

## Methods

### Study system and approach

**Fig 1:**
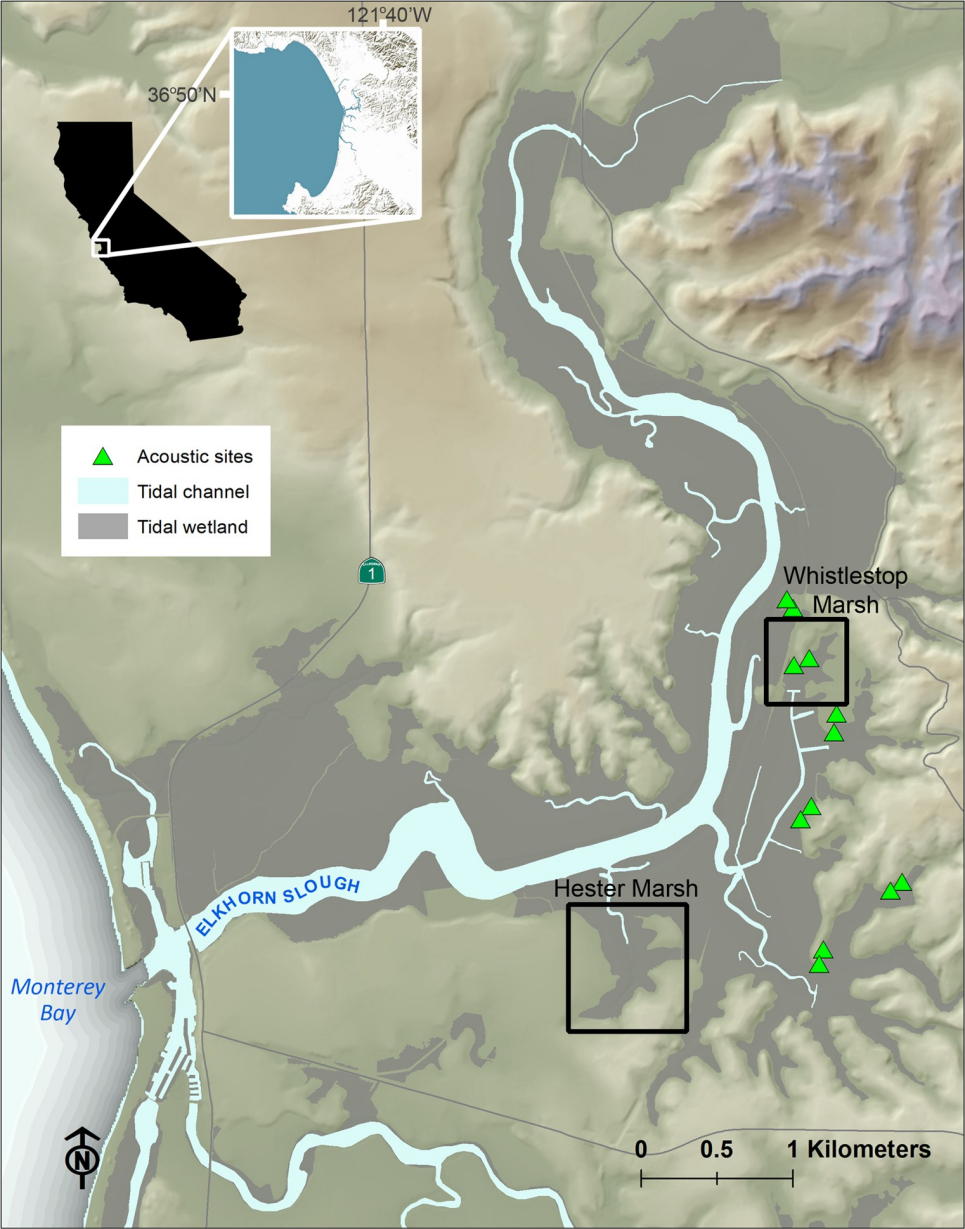
Location of Elkhorn Slough and restoration sites. The locations of the restoration sites at Hester and Whistlestop Marsh at Elkhorn Slough are shown, as well as the locations of the acoustic experiment. Image created using 10 m Digital Elevation Models from the USGS National Elevation Dataset.

The Elkhorn Slough estuary is located in the middle of Monterey Bay, central California, USA, in a watershed dominated by agriculture (Fig 1). The mean daily tidal range is about 1.6 m, with an annual maximum of 2.5 m. Salinity in the estuary averages 30–32 ppt year-round due to strong marine influence, although it can drop temporarily during heavy rainfall events. The climate is Mediterranean, with almost all rainfall occurring between October and May. Salt marshes at Elkhorn Slough are dominated by pickleweed (*Salicornia pacifica)*, which forms a virtual monoculture in mid-low elevations on the marsh plain. Other native marsh plants are found at the highest intertidal elevations, mostly between Mean Higher High Water and the King Tide line. The marsh-upland transition zone or ecotone is thus important for representation of native marsh diversity [26]. The grasslands landward of the King Tide line are highly invaded, often with large stands of tall forbs, including poison hemlock *(Conium maculatum*), mustards (*Brassica* spp.), and thistles (e.g. *Carduus pycnocepahulu*s, *Silybum marianum*).

**Fig 2:**
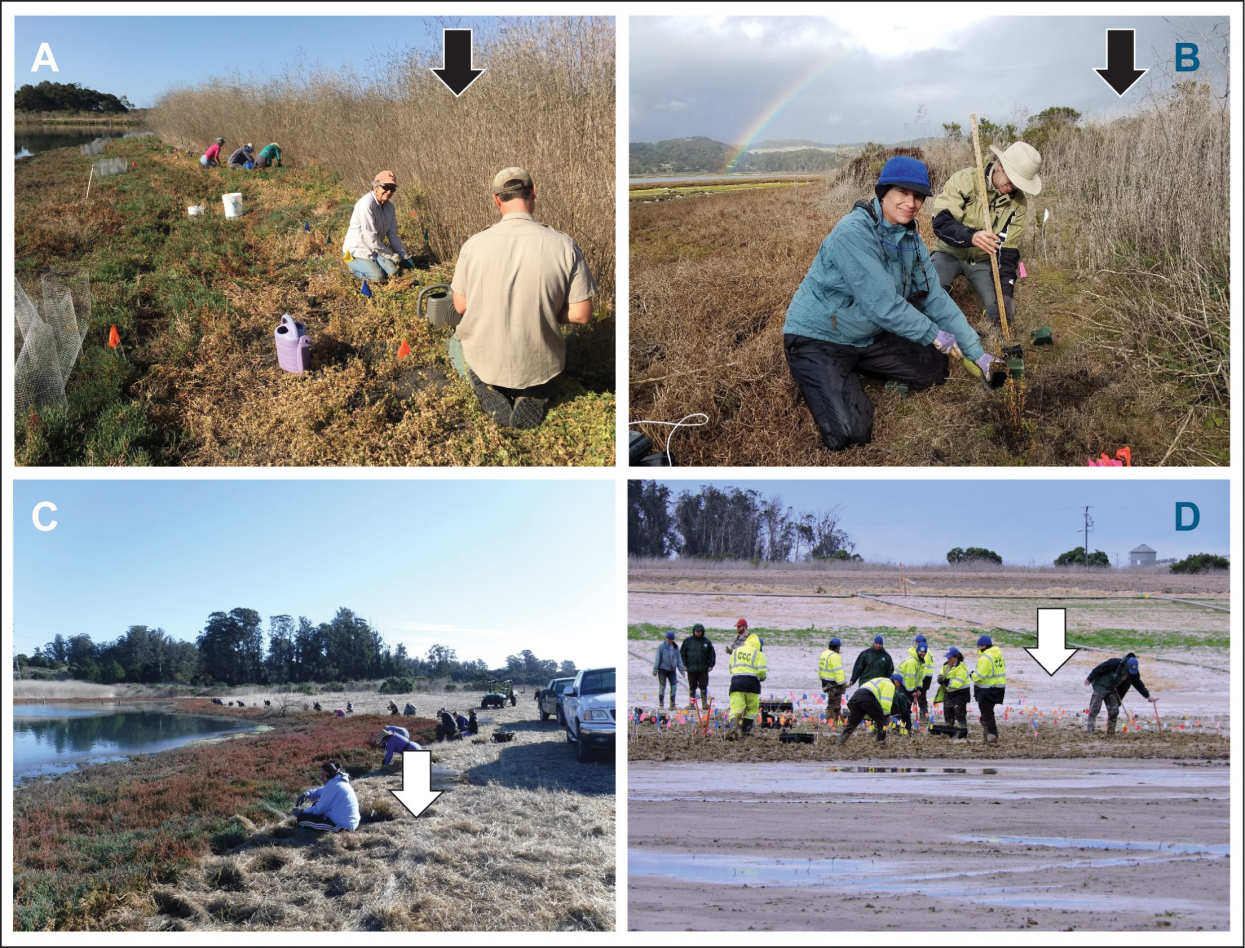
Landscape setting of study sites. (A) Whistlestop Marsh restoration planting in high marsh where tall exotic forbs (black arrow) are immediately landward of marsh; (B) Coyote Marsh experimental plantings for acoustic experiment in area similarly adjacent to exotic forbs (black arrow); (C) Whistlestop Marsh restoration planting in high marsh where high forbs have been mowed (white arrow) adjacent to marsh; (D) Hester Marsh restoration planting with freshly seeded grassland restoration immediately adjacent, with mostly bare cover (white arrow).

Elkhorn Slough has lost about half of the salt marsh area that was evident on maps 150 years ago, mostly due to diking and draining that occurred during the early 1900s [24]. In recent decades, natural tidal exchange has been restored to some of the marshes that formerly were diked. Whistlestop Marsh (Fig 1), on the Elkhorn Slough Reserve, is an example of such a restoration project. Full tidal exchange was restored in 2014, leading to a dramatically increased tidal range and dieback of upland vegetation that had encroached into marsh elevations during decades of diking. The Reserve planted greenhouse-grown high marsh plants into this bare zone to increase cover and marsh species diversity. Extensive stands of invasive upland forbs persist just above the King Tide line, such that canopy height jumps from low in the marsh to high in the adjacent grassland in many places (Fig 2A and 2B).

Diking and draining led to significant compaction of wetland soils at Elkhorn Slough, with substantial loss of elevation. When natural tidal exchange is restored to such marshes, most of the former marsh area is too low to sustain marsh vegetation, due to excessive inundation duration—all that remains is a narrow bathtub ring of marsh (Fig 2C). One restoration strategy to remedy subsidence due to diking is the placement of sediment to raise the elevation. This approach has recently been undertaken at the Reserve’s Hester Marsh (Fig 1). This 25-hectare site was built up with about 176,000 cubic meters of added sediment in 2018, such that most of the former marsh area is now above Mean Higher High Water. Recognizing that the site would initially be completely bare, and that recruitment of rarer high marsh species might be limited, the Reserve planted thousands of greenhouse grown high marsh plants into this area in January 2019. Because the surrounding area had been highly impacted by construction, including scraping of upland slopes to provide sediment and to create a gentle grade for future marsh migration, there were very few upland plants adjacent to the high marsh at that time (Fig 2D).

We monitored herbivory at these two major restoration projects on the Elkhorn Slough Reserve (as Reserve employees, three of the authors led these restoration projects and had permission from the Reserve). At Whistlestop Marsh, we used experimental restoration plantings designed to quantify and mitigate herbivore effects. At Hester Marsh, we predicted herbivory would be extremely low due to the limited terrestrial vegetation cover remaining after construction, and simply monitored restoration outcomes. We also carried out an experiment replicated at six sites across the Reserve to assess spatial variation in herbivore pressure and explore the potential for fear of predators to affect herbivory. Here, we have compiled these restoration experiments and monitoring efforts because they cumulatively shed light on the factors affecting variation in strength of top-down effects on restoration, and thus are most useful considered together. The approaches as well as major results of the four experiments are summarized in Table 1, and described in detail below.

**Table 1:** Overview of the four study components.

		Lessons learned	Camera trapping effort and detections	Upland forb cover	Herbivory	Plant survival	Stem length
Whistlestop Marsh	**Caging**	Herbivory rates are sometimes extremely high on restoration plantings *D, E, F, J)	Extensive trapping; many brush rabbits, some woodrats detected	High	High for uncaged plants	High in cages, low outside cages	Longer in cages
	**Mowing & caging**	Mowing adjacent terrestrial vegetation as well as caging effectively deters herbivory on restoration plantings (F)	Limited trapping in mowed areas only; rare deer detected	Low in mowed areas, high elsewhere	Low in mowed areas (caged & uncaged), high in uncaged areas without mowing	Not assessed	Not assessed
Hester Marsh	**Sediment addition restoration**	Herbivory is very low at new restoration site where earth-moving removed terrestrial vegetation (D, E, F, J, S)	Limited trapping; rare rabbits, deer detected	Very low	Very low	Very high for uncaged plants of all species	Not assessed
Six sites across the Reserve	**Site variation and acoustic experiment**	Herbivory rates are highly variable across sites and are correlated with rabbit abundance, but unaffected by predator playback treatment (F)	Extensive trapping; many rabbits and some woodrats detected but variable across sites	High	Variable by site, but no effect of predator calls	High across sites, no effect of predator calls	Variable and correlated with rabbit detections, but no effect of predator calls

*Plant species abbreviations: D—*Distichlis spicata*, E—*Extriplex californica*, F—*Frankenia salina*, J—*Jaumea carnosa*, S—*Spergularia macrotheca*

### Caging experiment

**Fig 3:**
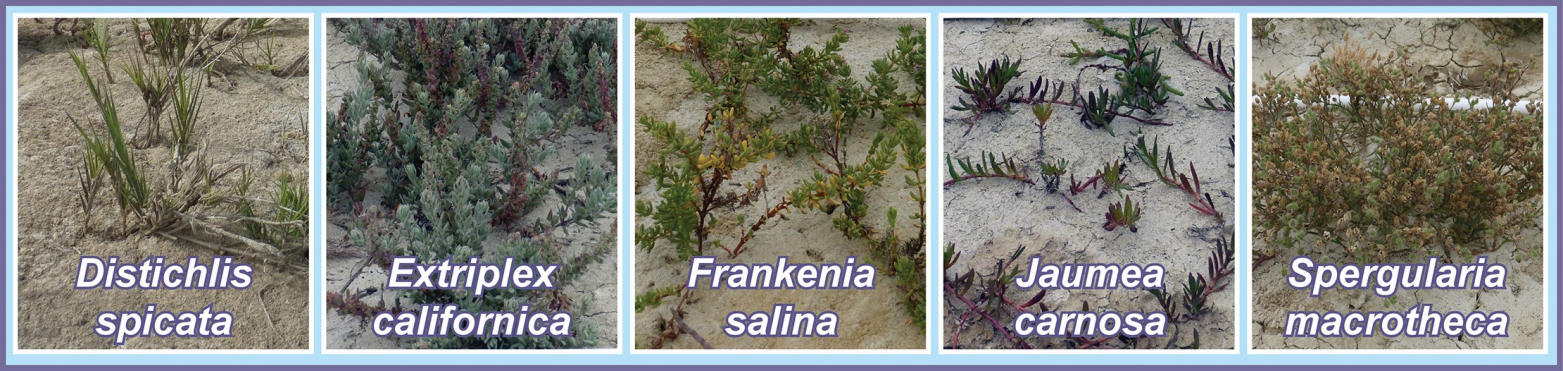
High marsh species used for restoration experiments. All five species were used in the Hester Marsh restoration experiment; other experiments used a subset of these species (see text for details).

**Fig 4:**
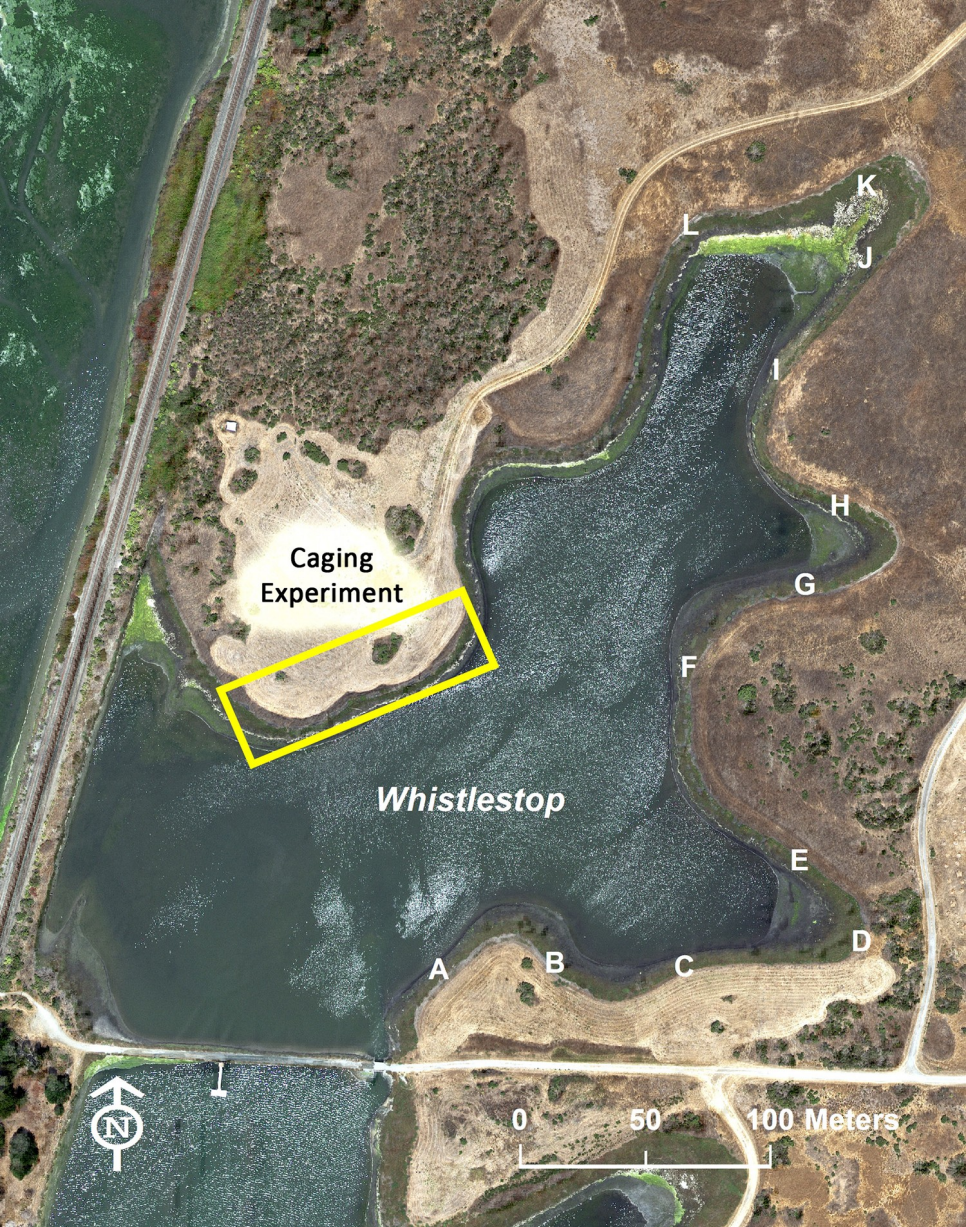
Locations of restoration with caging and mowing. The location of the caging experiments and the mowing experiment (lettered sites) at Whistlestop Marsh are shown. This photo is from USDA’s National Agriculture Imagery Program in 2018, the year following the experiments, when more extensive mowing was implemented in the caging experiment area and in areas A-D than during the period of the experiments. Salt marsh habitat appears as a narrow green “bathtub ring” adjacent to the water in this wetland that had been diked, with the majority of the historic marsh plain subsided too low to sustain marsh when tidal exchange was restored.

In Spring and Fall 2017, we initiated caging experiments to quantify the strength of herbivory and compare it among plant species. In Spring, we used two high marsh species, *Frankenia salina* and *Jaumea carnosa*. In Fall, we added two more species, *Distichlis spicata* and *Extriplex californica* (Fig 3). These experiments were conducted as part of a restoration effort at Whistlestop Marsh, where native diversity was very low after decades of artificially restricted tidal exchange. We established ten consecutive blocks in the upper ecotone on the northwestern shore (Fig 4), selecting areas with low or no marsh plant cover near the landward margin of the marsh. Plants were spaced 50 cm apart in blocks at two tidal elevations, either immediately landward of the upland border (where non-native upland plants grew) or 1 m seaward of this boundary to test whether plants closer to the upland would be more vulnerable to herbivory. All plant material was sourced from cuttings or seeds from the Elkhorn Slough watershed in 2016; plants were grown in the greenhouse in stubby containers or rose pots.

On 2 March 2017, we planted two *Frankenia* and two *Jaumea* individuals at two tidal elevations, in the upper and lower portions of each block (four plants per species in blocks measuring 1 × 3 m). We randomly caged half of the high and low plants of each species using 30 cm tall hardware cloth (0.6 cm mesh) cylinders 23 cm in diameter, held down by garden staples. Watering was not necessary because planting occurred during the rainy season. Plants were checked for signs of herbivory (truncated stems) on 11 April and a final survey on 6 June, when we also recorded plants with green tissue as alive and measured the length of the longest stem to the nearest 0.5 cm.

On 1 November 2017, we repeated the experiment, planting two individuals of all four genera (*Frankenia*, *Jaumea*, *Distichlis*, *Extriplex*) in the upper and lower portions of each block (four plants per species in each 1 × 4 m block). Planting was conducted by restoration volunteers. We used a slightly different but equally effective cage design (45 cm tall, 29 cm diameter cylinders of 1.2 cm mesh hardware cloth), and plants were watered immediately after transplanting due to dry soil conditions. Plants were assessed as described above on 14 November 2017 and 9 February 2018. Four camera traps were also installed on planting day, with trail cameras aimed at uncaged plants in different blocks. The cameras were retrieved three weeks later and photographs examined to determine which herbivores ate the plants, and how quickly.

To evaluate treatment effects on survival and final stem length we used separate generalized linear models (GLMs) for the spring and fall experiments. We used a binomial GLM to evaluate survival, including the main effects species, caging treatment (caged vs. uncaged), tidal elevation (high vs. low), plus the caging treatment × tidal elevation interaction. We used the same predictors in a GLM evaluating final stem length, but data sets were strongly zero-enriched and could not be transformed to achieve normality. We therefore dropped plants that were entirely consumed from the data sets before analysis. For the spring experiment we followed the same procedure and also applied a square-root transformation to improve normality of the data. Block was not included as a random effect in survival or stem length analyses because blocks were incomplete. We used R version 3.6.3 [29] to build generalized linear models (GLMs) and the Anova function from the car package [30] to generate Type II *P*-values. To visualize results for some analyses, we used the ggplot2 [31] and ggpubr [32] packages.

### Mowing and caging experiment

The purpose of this experiment was to test whether mowing exotic forb cover adjacent to the marsh deters herbivores, and to assess whether large cage exclosures can protect restoration plantings. In December 2017, Elkhorn Slough Reserve staff mowed the tall forbs immediately adjacent to the southeastern portion of Whistlestop Marsh in a strip about 200 m long (Fig 2C). All standing biomass in the western portion was cleared to the ground in a swath 5 m wide (Fig 4A and 4B). Towards the east, mowers avoided scattered large shrubs and interspersed forbs (Fig 4C), and the mowed swath narrowed to ~ 3 m at the eastern end of the shoreline (Fig 4D); beyond that mowing stopped at a large cluster of shrubs. In the contiguous section of shoreline running north (Fig 4E–4L), forbs were already recolonizing a 2 m wide swath that had been mowed in November 2017 to allow access to an adjacent experiment. The shoreline of Whistlestop Marsh at this point thus was a mosaic of mowing intensity, allowing for comparisons of herbivory across this gradient. (The photo in Fig 4 was taken in the year following this experiment, when width of mowed swath and intensity of mowing had increased in both southern and northern areas of the marsh.)

On 6 December, 16 December, and 18 December 2017, Elkhorn Slough Reserve staff organized three community restoration events to outplant over 1000 greenhouse-grown, rose-pot-sized *Frankenia salina* seedlings along the landward marsh edge of the Whistlestop Marsh shoreline, spacing plants about 30 cm apart. In the most thoroughly mowed western portion A (Fig 4), small individual cages (30 cm tall, 10 cm in diameter) were placed around 46 plants, and 46 adjacent plants were flagged for a cage/no cage comparison. This allowed strength of herbivory in this thoroughly mowed area to be compared to earlier results from March and November Caging Experiments (above) in the unmowed area.

Where the mowed swath was narrower and large shrubs still provided some cover for herbivores, three large cage exclosures were constructed (one each in areas, Fig 4B–4D). The exclosures were constructed from chicken wire and wooden stakes and measured approximately 7.5 m long by 0.5 m wide by 0.5 m tall. Seven plants inside each fenced area were flagged, as were seven plants immediately outside the fenced area (three on one side, four on the other). This allowed us to test the efficacy of large cage exclosures, and assess strength of herbivory in areas with varying mowing intensity. A camera trap was placed near the uncaged flagged plants in each of these blocks for two weeks following planting.

In each area E-L, seven uncaged *Frankenia salina* seedlings were planted and flagged to compare the strength of herbivory along this section of shoreline (where the mowed swath was narrower and mowing was less recent) to the southeastern shoreline A-C (where mowing was more recent and thorough).

The restoration plantings were assessed on 10 January 2018 for signs of herbivory, when camera traps were also retrieved and photos examined. To compare areas with higher mowing intensity (A-C) to areas with lower mowing intensity (D-L) we calculated the average proportion of plants with signs of herbivory for each block and conducted a T-test using block as replicate. Survival was not assessed due to the relatively short duration of the experiment.

### Hester restoration

In January 2019, the Elkhorn Slough Reserve planted about 17,000 marsh plants at the otherwise bare Hester Marsh restoration site. The plants were placed in six blocks at the landward margin of the marsh, each one about 30 m long and 35 m wide and spanning the uppermost 30 cm of the tidally inundated area (from the King Tide line to 30 cm below that). Planted blocks alternated with unplanted blocks of similar size. Five different high marsh species grown in local greenhouses from watershed seeds or cuttings were planted in each block (*Distichlis spicata*, *Extriplex californica*, *Frankenia salina*, *Jaumea carnosa*, *Spergularia macrotheca*; Fig 3). The uplands adjacent to the marsh plantings were nearly bare at the time of planting. They had been seeded or planted with small plants in December 2019, but the dominant cover was still bare ground and canopy height was very low (Fig 2D). Given the lack of upland cover, we assumed that terrestrial herbivore abundance would be low and the restoration plantings were not caged. All plants were planted between 7–24 January 2019.

In each of the six planted blocks, we flagged 18 plants near the landward end of the block, and 18 plants near the seaward end, for each of the five species. We monitored these 216 plants per species (18 plants x 2 elevations x 6 blocks) for survival and herbivory approximately 3 weeks (11 February), 14 weeks (22 April), 19 weeks (3 June) and 23 weeks (1 July) after planting. Survival was defined as the presence of green tissue on stems; freshly clipped stems were attributed to herbivory. On the first monitoring check we discovered that the wrong plant species was flagged in two places and these individuals were dropped from the data set, so that the total number of monitored plants was 215 (instead of 216) for *Extriplex* and *Frankenia*. We intended to compare survival among species, elevations and blocks, but survival was so universally high that no statistics were conducted. In July 2019, we set two camera traps near the flagged plants at the landward edge of two planted blocks to determine which animals frequented the area, and retrieved them after 15 days.

### Site variation and acoustic experiment

A main objective of this study was to compare herbivory rates and herbivore abundance among sites to characterize spatial variation in these factors, and to determine the strength of their correlation (i.e. is more marsh plant tissue eaten at sites where more herbivores are detected by camera traps). An additional objective was to determine whether the playback of predator calls could discourage herbivory in restoration areas by instilling fear in rabbits and other terrestrial herbivores.

We selected six sites on the Elkhorn Slough Reserve (Fig 1). Each had a relatively narrow band of marsh resulting from a history of diking. At each site, we attempted to locate two areas with similarly dense stands of high forbs adjacent to the marsh, with about 100 m separation between paired locations (average distance between paired locations at each site = 109 m, range 73–206 m). One area was randomly assigned to the predator treatment, the other to the control (see below).

At each of the paired treatment locations (two per site), ten greenhouse grown *Frankenia salina* seedlings were planted in a row. Plants were spaced at 10 cm intervals about 20 cm seaward of the upland-marsh boundary and were not caged. A custom-designed, battery powered loudspeaker was placed in the upland vegetation about 3 m landward of the plantings, positioned about 1 m above the ground. A trail camera (Bushnell Trophy Cam) was placed 1.1–1.6 m from the center of the planted row, pointing toward it, 0.55–0.75 m above the ground.

At the locations randomly assigned to the predator treatment, the loudspeaker played vocalizations of coyotes (*Canis latrans*) and great horned owls (*Bubus virginianus*). At the areas assigned to the control treatment, the loudspeaker played vocalizations of snowy egrets (*Egretta thula*) and marbled godwits (*Limosa fedoa*). All four of these animal species are common on the Elkhorn Slough Reserve. Multiple vocalizations (2–5) were used per species and the order was randomized. The loudspeakers ran continuously, day and night, and were programmed to broadcast predator or control vocalizations 40% of the time (thus remaining silent 60% of the time).

The experiment was initiated on 22 February 2018, when seedlings were planted and loudspeakers and cameras were deployed, and concluded on 23 March 2018. We visited sites approximately weekly to maintain loudspeakers and camera traps (swapping batteries and troubleshooting as needed), and to survey plants for signs of herbivory. On two of the four weekly visits, one out of six of the predatory playback loudspeakers was no longer playing when we arrived (at one location one week, another location another week). One of the six control playback speakers was not playing when we arrived at two consecutive weeks at one of the locations. Without knowing how long before the visit they had stopped working, we cannot assess how much of the time the treatments were being applied as intended. The technical issues encountered with speakers indicate the treatments were not applied as consistently as intended, but were applied appropriately most of the time at most locations. Camera trap photos were sorted and detections of all animals quantified per site, with detections considered independent if spaced >3 min from previous detection. Plant survival and maximum stem length were also assessed at the end of the experiment, and final herbivory status was assigned based on signs of consumption at any point during the experiment.

We conducted a linear regression of consumer abundance vs. change in plant stem length, using each location with a camera and planting (two per site) as replicate. To evaluate whether predator calls discouraged herbivory on restoration plantings, we used the lme4 package to build a generalized linear mixed model (GLMM, [33]) with playback treatment (predator calls vs. shorebird calls) as a fixed effect, and location nested within site as a random effect. The data set was strongly non-normal because heavy herbivory in some locations led to many plants with final stem lengths of zero; however, we wished to retain these data in the analysis because dropping zeroes eliminated all the data for certain locations. We therefore added 1 to all stem length values, and specified a gamma distribution with a log link function in the GLMM to better accommodate the shape of the data (confirmed using model diagnostic plots). We also analyzed plant survival by playback treatment using a binomial GLMM with location nested within site as a random effect. Lastly, we built a GLM including only site (Fig 1) as a fixed effect to evaluate variability in herbivory around Elkhorn Slough.

## Results

### Caging experiments

**Fig 5:**
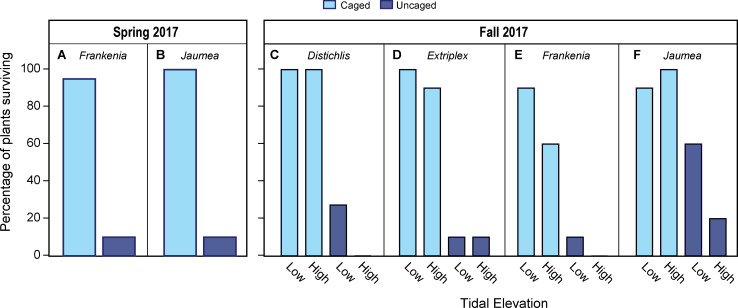
Final survival in the Spring 2017 and Fall 2017 experimental restoration. Survival by caging treatment (the only significant predictor) is shown in (A) for *Frankenia* and (B) for *Jaumea* in the Spring 2017 experiment; bars show survival for both tidal elevations combined. Survival by caging treatment and tidal elevation (both significant predictors of survival) is shown in (C) for *Distichlis*, (D) for *Extriplex*, (E) for *Frankenia*, and (F) for *Jaumea* in the Fall 2017 experiment. By the final survey date for each experiment, very few uncaged plants remained alive, while most caged plants remained alive.

In both the Spring and Fall 2017 experiments, caging treatment was a highly significant predictor of survival, with herbivores rapidly consuming uncaged restoration plantings (Tables 2 and 3; Fig 5). At least 95% of uncaged plantings were grazed by herbivores in both experiments, but herbivory occurred more slowly in the Spring than in the Fall experiment. On the first monitoring survey in Spring many uncaged plants were still intact, while most uncaged plants had already been consumed in the Fall experiment, even though the time period between planting and the first survey was shorter in Fall (Table 2). In both experiments, some *Jaumea* that were scored as dead on the first survey (no green tissue present) were scored as alive on the final survey, showing the potential for plants to recover from herbivory.

**Table 2:** Numbers of plants surviving in Spring and Fall 2017 restoration.

			2 Mar	11 Apr	6 Jun
		Genus	Planting day	First survey	Final survey
**Spring 2017**		** **			
**(A)**	Uncaged	*Frankenia*	20	20	2
		*Jaumea*	20	14	2
**(B)**	Caged	*Frankenia*	20	19	19
		*Jaumea*	20	19	20
			1 Nov	14 Nov	9 Feb
		Genus	Planting day	First survey	Final survey
**Fall 2017**		** **			
**(C)**	Uncaged	*Distichlis*	21	4	3
		*Extriplex*	20	1	2
		*Frankenia*	20	1	1
		*Jaumea*	20	0	8
**(D)**	Caged	*Distichlis*	18	18	18
		*Extriplex*	20	20	19
		*Frankenia*	20	20	15
		*Jaumea*	20	20	19

By the final surveys, very few uncaged plants remained alive (A, C) while most caged plants remained alive (B, D).

**Table 3:** GLM results for caging effects on survival and stem length in restoration plantings.

		Predictor	Sum Sq.	Df	F value	*P*-value
**Spring 2017 caging experiment**						
**(A)**	Survival	Genus	0.22	1	0.29	0.595
		Caging treatment	75.43	1	97.22	**< 0.001**
		Tidal elevation	0.22	1	0.28	0.595
		Caging treatment × Tidal elevation	1.19	1	1.54	0.219
		Residuals	58.19	75	NA	NA
**(B)**	Stem length	Genus	3.42	1	6.86	**0.011**
		Caging treatment	135.55	1	271.87	**< 0.001**
		Tidal elevation	0.64	1	1.29	0.261
		Caging treatment × Tidal elevation	1.95	1	3.92	0.053
		Residuals	27.42	55	NA	*NA*
**Fall 2017 caging experiment**						
**(C)**	Survival	Genus	15.86	3	3.22	**0.025**
		Caging treatment	111.54	1	67.90	**< 0.001**
		Tidal elevation	7.59	1	4.62	**0.033**
		Caging treatment × Tidal elevation	0.26	1	0.16	0.692
		Residuals	249.68	152	NA	NA
**(D)**	Stem length	Genus	2030.75	3	19.15	**< 0.001**
		Caging treatment	1058.26	1	29.93	**< 0.001**
		Tidal elevation	1.43	1	0.04	0.841
		Caging treatment × Tidal elevation	231.75	1	6.56	**0.012**
		Residuals	2757.62	78	NA	NA

Generalized linear model results evaluating effects of species, caging treatment, and tidal elevation on survival and final stem length in two caging experiments. Spring 2017 results are shown in (A) for survival, and (B) for final stem length. Fall 2017 results are shown in (C) for survival, and (D) for final stem length.

**Fig 6:**
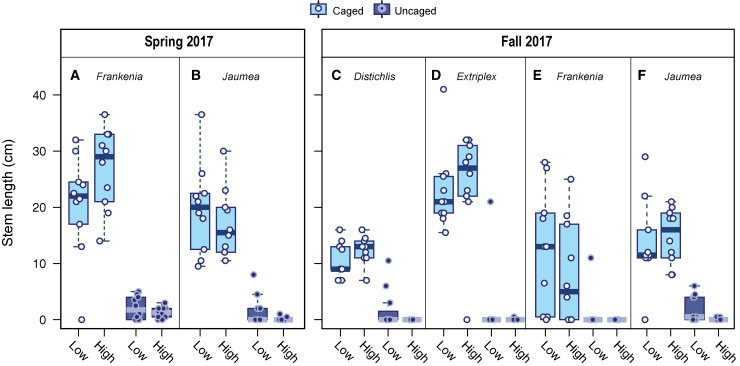
Final stem length by caging treatment in restoration plantings. Maximum stem lengths from the final survey are shown (A) for *Frankenia* and (B) for *Jaumea* in the Spring 2017 experiment, and (C) for *Distichlis*, (D) *Extriplex*, (E) *Frankenia*, and (F) *Jaumea* in the Fall 2018 Experiment. There was no significant difference in plant size by treatment on planting day for either experiment. The figure includes plants that were dropped prior to statistical analysis (those with a final stem length of zero).

Maximum stem length showed clear patterns across treatments at the end of both experiments. Genus had a strong influence on final stem length (Table 3B and 3D), likely related to the different growth forms of the four species. Caging treatment had a strong effect on final stem length for all species in both experiments (Fig 6). The caging treatment × tidal elevation interaction was marginally significant in the Spring experiment (Table 3B) and significant in the Fall experiment (Table 3D), likely reflecting a pattern of longer stems on uncaged plants at low tidal elevation.

**Fig 7:**
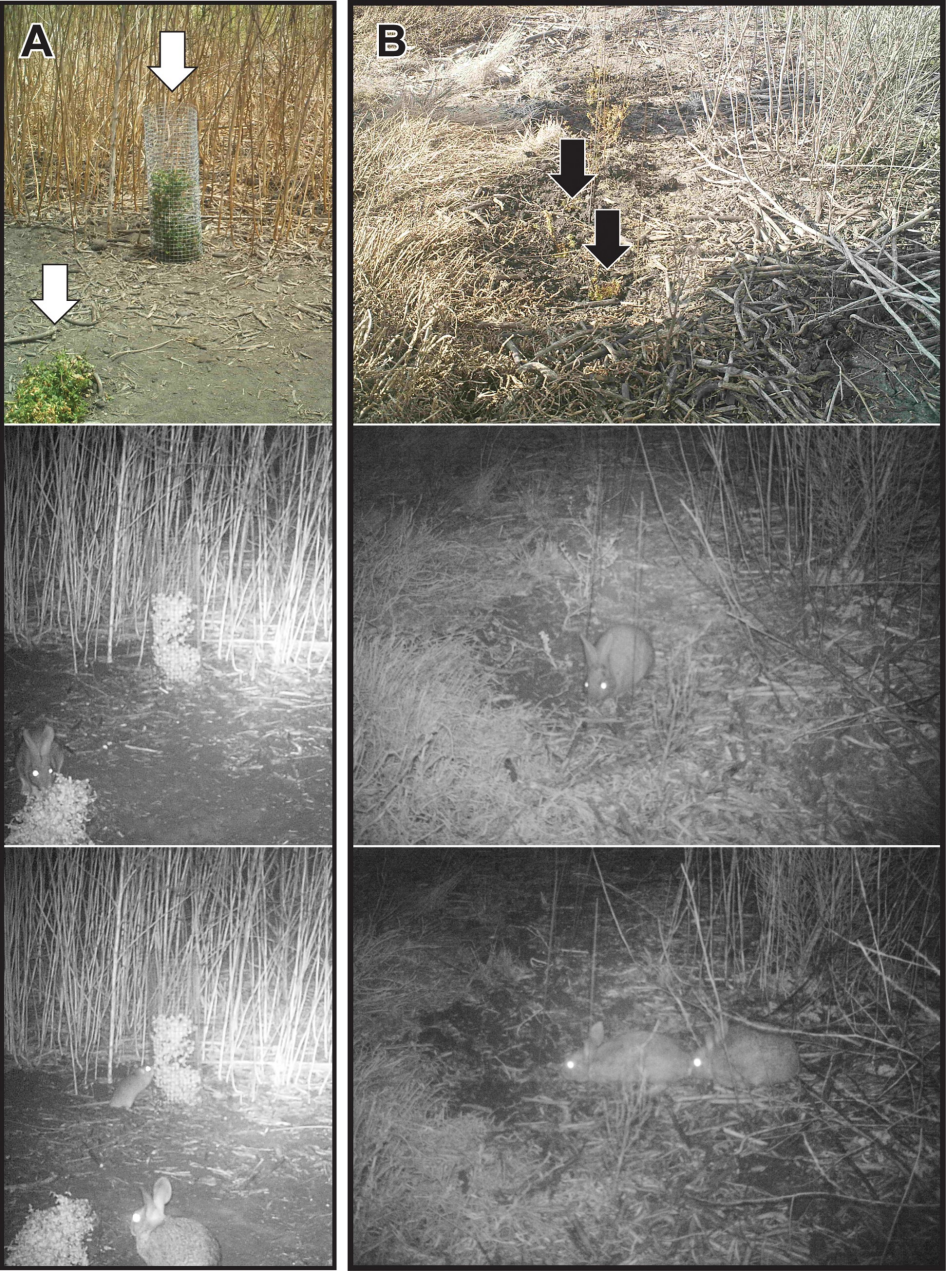
Herbivores on marsh plants at Whistlestop in Elkhorn Slough. *Frankenia* outplants in (A) the caging experiment, and (B) the acoustic experiment at Whistlestop (seedlings indicated by arrows). The top row shows the plantings by day; the lower rows show herbivores photographed at the same locations by night (mostly brush rabbits, but a woodrat was also observed eating a restoration planting—see the lower left photo).

The four trail cameras aimed at uncaged plants in the experiment revealed that brush rabbits were by far the most frequent consumers of plants (Fig 7). In total, there were 250 photos of brush rabbits (*Sylvilagus bachmani*); 5 photos of dusky-footed woodrats (*Neotoma fuscipes*); 9 photos of birds, and zero photos of other animals. Brush rabbits were observed consuming the restoration plants in many of the photos and woodrats were occasionally observed eating the plants. No birds were observed consuming the plants. Almost all the animals visited the restoration plantings under cover of darkness. The uncaged restoration plants in view of each camera generally suffered heavy herbivory within four days of planting.

### Mowing and caging experiment

**Fig 8:**
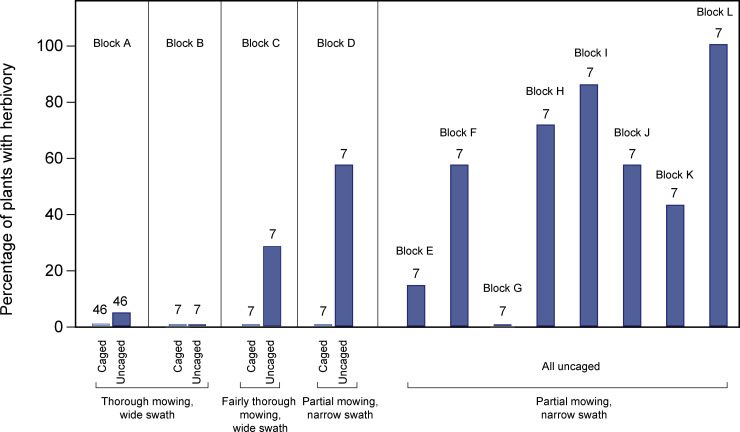
Herbivory rates in restoration plants in areas with different mowing intensity. Block lettering at the top of panels corresponds to the locations in Fig 4. *Frankenia salina* plants were transplanted to Whistlestop in December 2017 and assessed for herbivory in January 2018. Numbers above bars indicate the number of plants in each block and treatment. Only uncaged individuals were planted in blocks E-L.

The mowing experiment revealed varying levels of herbivory that corresponded with mowing intensity (Fig 8). In the blocks where mowing was thorough in a wide swath adjacent to the marsh (Fig 4A and 4B), herbivory rates were very low. In block A, 2 out of 46 uncaged plants showed signs of light herbivory, while all 46 individually caged plants remained intact. In block B, there were no signs of herbivory on the 7 plants inside the large cage exclosure or the 7 plants immediately outside of it. As mowing intensity declined in blocks C and D, more herbivory was observed, with 2 and 4 out of 7 uncaged plants showing signs of herbivory, respectively. Photos from camera traps aimed at the mowed, uncaged zones for two weeks during the experimental period detected black-tailed deer (*Odocoileus hemionus columbianus*), raccoons (*Procyon lotor*), and a frog (*Pseudacris triseriata*), but no rabbits or woodrats.

Along the shoreline of the wetland where upland vegetation was still partially cleared from earlier mowing in a narrow swath, we found highly variable herbivory rates on the 7 plants placed in various areas (Fig 4E–4L)—a range from 0 to 7 of the plants showed signs of herbivory (Fig 8).

The average proportion of plants with signs of herbivory in the blocks with high mowing intensity (A-C) was 11% vs. 54% in the blocks with low mowing intensity (D-L), and the difference between these groups was statistically significant in a T-test (*P* = 0.016).

### Hester restoration

Survival of the flagged high marsh species planted in blocks at the restoration site was extremely high. After 23 weeks, 100% of flagged plants were alive for four species; all 216 flagged plants per species were still alive for *Jaumea*, *Distichlis*, and *Spergularia*, and all 215 flagged *Frankenia* plants were still alive (this group had one less flagged plant due to an initial identification error). For *Extriplex*, one of the 215 flagged plants was dead at 19 weeks; no additional mortality occurred at 23 weeks, so 99.5% of flagged plants were alive for this species. We found signs of herbivory (freshly clipped stems) on plants of only one species; nine *Spergularia* individuals had signs of herbivory at the 19 week check, and three at the 23 week check.

We detected very few animals over the 15 days camera traps were deployed at this restoration site. Animals were only detected in darkness. One camera detected a single brush rabbit on two different nights, a deer, and a raccoon. The other camera detected one brush rabbit once. No other animals were detected with the camera traps, though Canada geese (*Branta canadensis*) were often seen in the area during daytime surveys, and their excrement and tracks were found near the restoration plantings. Especially in the first weeks after planting, we noted fresh herbivory on *Spergularia* plantings in areas geese frequented, with the small plants ripped out of the ground, but mostly the geese appeared to forage in the adjacent newly planted grassland.

### Site variation and acoustic experiment

Camera traps allowed us to characterize the animal communities at the six marsh sites over a period of 1 month. Brush rabbits were by far the most common animal observed (Fig 7B), with 470 total detections during the experiment for all 12 cameras combined. Per camera, there were an average of 39 rabbit detections with high variation (range from 0–108). The second most common animals were dusky-footed woodrats (108 total detections, 8 per camera on average, range of 0–32), followed by skunks (*Mephitis mephitis*) (16 total detections, 1 on average, range 0–6). Over the course of the month all other animals were detected 10 or fewer times across all 12 cameras. These included black-tailed deer, raccoons, bobcats (*Lynx rufus*), coyotes and owls (*Bubus* or *Tyto*). Results from the acoustic experiment indicate that rabbit and rat detection rates were not significantly different between locations playing predator calls vs. shorebird calls, although they were higher in the latter.

**Fig 9:**
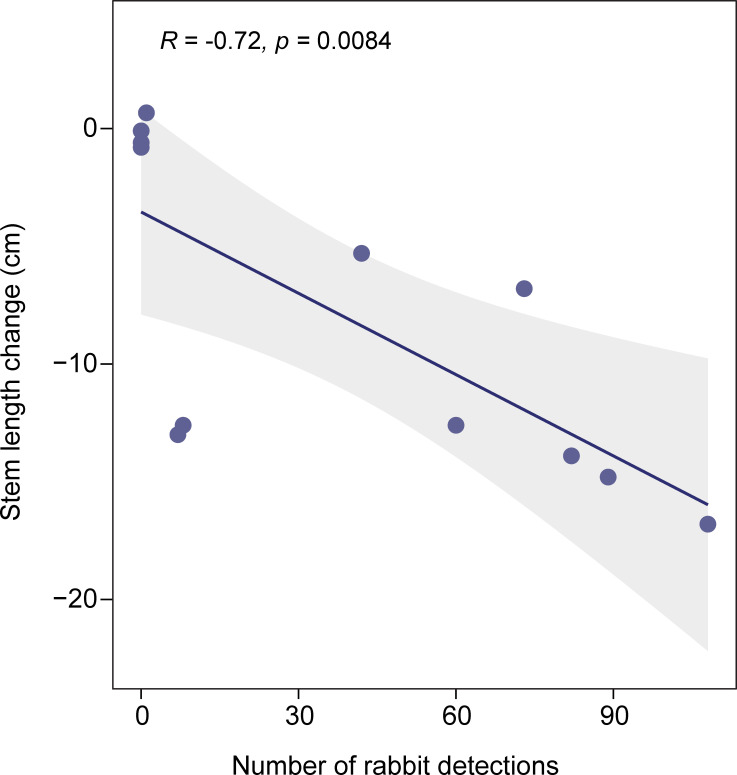
Relationship between rabbit detections and restoration plantings. Rabbit detections were summed over the month-long experiment. Maximum stem length at the end of the experiment was subtracted from that at the beginning of the experiment, and averaged across the ten plants per location. The 12 locations (two per site, Fig 1) were used as replicates for a simple regression analysis.

**Fig 10:**
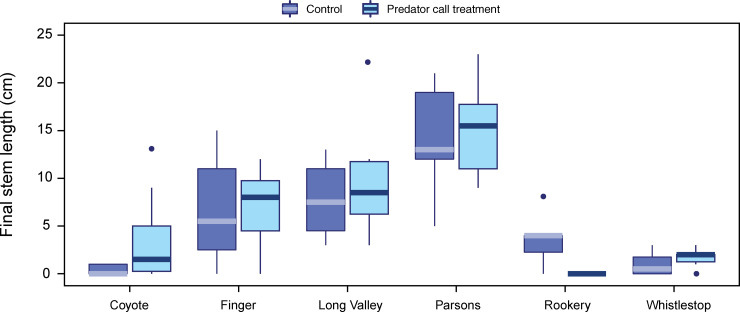
Stem length of restoration plantings by site and playback treatment. Length of the longest stem per plant at the final assessment was used as the response variable. See Fig 1 for site locations.

The length of the longest stem for the ten *Frankenia* plants at each site generally decreased over the month-long experiment; the plants appeared physically stressed in addition to the signs of herbivory. Change in stem length was negatively correlated with rabbit detections at the location (Fig 9); this relationship was slightly weaker with total potential consumers (rabbits + woodrats). Stem length differed significantly among sites (gamma GLM: likelihood ratio Chi-square = 71.632, df = 5, *P* < 0.0001). However, stem length did not differ between playback treatments, although stems were longer in the predator call treatment at five out of six sites (gamma GLMM: likelihood ratio Chi-square < 0.001, df = 1, *P* = 0.9766; Fig 10). The prevalence of herbivory did not differ between playback treatments, with 62.7% of plants in the control treatment and 61.6% in the predator treatment exhibiting herbivory at some point during the experiment. More than 93% of plants survived to the end of the experiment, with no effect of playback treatment on survival (binomial GLMM: likelihood ratio Chi-square = 0.0168, df = 1, *P* = 0.8969).

## Discussion

### Importance of top-down effects in California salt marsh

Around the world, consumers have been shown to exert strong influences on vegetated habitats, including on coastal foundation species [34–36]. However, there have been surprisingly few studies of top-down effects on salt marshes of the North American Pacific coast. We found only four published papers examining consumer effects on marsh vegetation along this entire coastline; while our literature search may have missed some studies, it is clear there have not been many. Two studies documented scale insect effects on *Spartina* in southern California marshes [37, 38]. One experimental investigation detected subtle, interactive effects of snails and crabs on *Salicornia* in another Southern California marsh [39]. Finally, a national investigation that included four Pacific coast marshes reported a significant negative relationship between crab burrows and marsh cover at one Oregon and one California estuary [25]. In contrast to this low number of publications, there are many studies examining the effects of bottom-up drivers such as inundation and salinity on Pacific coast marsh vegetation [e.g. 40–43].

We demonstrated the potential for very strong top-down effects in a California salt marsh; for example, in our first experiment, only 10% of uncaged plants survived, while at least 95% of caged ones did. At least for small plants of the four high marsh species we examined, herbivores may have a very significant effect on survival, and thus potentially on distribution and abundance of these species. Given that California marshes are dominated by pickleweed (*Salicornia pacifica*), and high marsh species are rare at estuaries such as Elkhorn Slough even in the marsh-upland ecotone where they are most abundant [26], this means that consumers may decrease marsh community diversity by preventing seedlings of rarer species from establishing.

The dominant herbivores we documented in this marsh system were brush rabbits. Lagomorphs have been reported from other salt marsh ecosystems, including a specialized marsh rabbit in the southeastern United States [44]. However, strong effects of lagomorphs on marsh vegetation have previously been documented in only one location—an island in the Wadden Sea in Northern Europe [45–47]. At Elkhorn Slough, the second most common herbivore we detected in the marsh was a rodent, the dusky-footed woodrat. Rodents are well known from other marshes, such as the marsh rice rat in the southeastern United States [48]. Yet we found only three systems where rodents are reported to have strong effects on marsh vegetation—mice and voles in olighaline marshes of the northeastern US [49], nutria in oligohaline marshes of the southeastern US [50] and guinea pigs in Argentina [22, 51]. Since lagomorphs and rodents are common in terrestrial habitats adjacent to marshes, no doubt they are influencing marsh vegetation in many more places, where such effects can be detected in future experiments.

### Dynamics at the marine/terrestrial ecotone

Ecotones are transitions between two habitat types, and often have particularly high diversity and concentrated animal populations [52, 53]. Marshes are at the landward edge of estuaries, adjacent to upland habitats. Salt marshes can thus be affected by the adjacent terrestrial habitat and consumers coming from there, including guinea pigs [54], rabbits and geese [47] and cattle [23]. The high marsh that we focused on in California is an ecotone between salt marsh and grassland habitat [26]. Previous studies in this system found that rodents move back and forth between the marsh and grassland seasonally; voles forage in the marsh during its peak growing season in the dry summer, while grasslands are brown, and then forage in the green grasslands in the rainy winter when the marsh is dormant [55, 56]. These studies did not examine effects of the rodents on vegetation, but make clear that both habitat types must be considered to understand drivers of vegetation in this transition zone. Thus to understand processes in ecotones, a landscape scale is most effective [57].

We detected strong spatial variability in abundance of consumers and concurrently in their consumption of high marsh plants. At least some of this variation appears to be the result of the structure of the grassland vegetation adjacent to the marsh. The stark contrast in consumer pressure between our two restoration sites highlights this: the site with extensive stands of tall exotic forbs adjacent to the marsh typically had 5–15% survival of uncaged restoration plantings, while the site with little adjacent upland vegetation due to recent construction had nearly 100% survival. The effects of exotic plants on grasslands are well known [58, 59], but our results highlight that the influence of these exotic forbs reverberates into the adjacent habitat, affecting plants seaward of the king tide line in the marsh by providing cover to consumers. Thus a landscape approach is critical for recognizing linkages between terrestrial and marine vegetation. The vegetation in these ecosystems is connected through shared consumers—and their predators—that move between them.

As a part of a landscape approach to understanding dynamics of the high marsh, we recommend camera trapping as an effective tool for characterizing herbivores. We were able to determine that the most common herbivores on high marsh vegetation were brush rabbits, and found a strong correlation between their abundance and growth of restoration plantings across sites. Of course, tiny herbivores such as snails or insects would not be detected by this method. But where herbivores are vertebrates, camera trapping can be useful for assessing relative abundance of herbivores, and how this varies with environmental context such as vegetation type or adjacent land uses. Such data can inform site selection or habitat management strategies for restoration projects.

### Restoration applications

Wetland restoration projects provide remarkable opportunities to understand vegetation dynamics through rigorous science, and thus to inform success of future restoration projects [5]. Salt marsh ecology and restoration projects typically focus on bottom-up processes in design and monitoring [7, 8]. Since marsh distribution, abundance and diversity are so closely linked to tidal elevation and inundation, an emphasis on physical parameters is justified. However, our results make clear that practitioners also need to consider top-down factors in restoration: we found alarmingly high rates of herbivory at one of our restoration sites, significantly affecting restoration success.

Seminal papers have highlighted the conceptual importance of considering consumers in marsh conservation [34, 36]. However, to date there have been very few published studies examining consumer effects on marsh vegetation at actual restoration sites. At a marsh restoration site in southern California, a scale insect outbreak caused some damage to *Spartina* [37]. Another southern California study examined the role of snails and crabs affecting marsh dynamics at a restored site; consumers had strong effects on algae and sediment properties, not on marsh vegetation itself [39]. At a restored marsh in Maine, superabundant snails grazed restoration plantings to the ground [60]. In China, failure of marsh restoration has been attributed to crabs [61, 62]. These are the only examples of top-down effects at marsh restoration sites we found in the literature, so our relatively modest investigation is nevertheless the most comprehensive to date, and the first documenting terrestrial mammal effects on marsh restoration success.

While raising concern about consumer effects on salt marsh restoration, our study also highlights strategies for reducing impacts. Most importantly, we recommend a landscape perspective: the adjacent terrestrial vegetation should be considered where high marsh species are vulnerable to terrestrial herbivores. In some cases, herbivory impacts can be minimized by prioritizing restoration areas adjacent to habitat lacking sufficient cover to foster abundant herbivores. For instance, at Elkhorn Slough, sites adjacent to native-dominated grasslands could be prioritized, since these are likely to harbor many fewer rabbits than tall, dense stands of exotic forbs. Alternatively, if restoration is slated to occur in marshes adjacent to exotic forbs, these can be managed in the first years following restoration, to reduce herbivore abundance. We were able to reduce herbivory from a high rate to near zero simply by mowing a swath just landward of the marsh. In addition to these landscape approaches, more targeted actions can also be taken, such as caging. We found individual cages around plants were highly effective, but they can only be used for weeks to months for most fast growing plants, which will then outgrow them. We also found larger cage exclosures to be effective; these may represent a better approach for large restoration projects, but require frequent maintenance. Another approach that may be effective in some systems is to select species less prone to herbivory, to use at restoration sites known to have high herbivore abundance, but this may limit vegetation diversity. We found high herbivory on all four species examined, but theoretically this is a worthwhile approach to consider elsewhere.

We also recommend continuing to explore the potential for predators on the herbivores to affect marsh restoration. Our experiment with predator playbacks yielded suggestive, but not compelling results for our system. However, the concept of harnessing fear of predators to enhance restoration success remains intriguing and is worth further examination. Indeed, fear of predators as a management tool has garnered increasing attention in terrestrial conservation applications [63, 64], and we suggest that such approaches may be similarly fruitful in transition habitats such as salt marshes. Insights from terrestrial applications suggest that the combination of multiple predator cues (e.g., auditory, olfactory, and visual) may increase the effectiveness of predator manipulations, providing a valuable starting point for further applications to marsh restoration. Actual predator abundance can also be manipulated. For instance, the Elkhorn Slough Reserve has recently installed perches for raptors and owls near its large marsh restoration site; the impact of such perches on marsh restoration could be formally quantified with large-scale experiments. In many areas, populations of predators have been reduced by human activities [65]. Restoring marsh communities vulnerable to terrestrial herbivory may benefit from concurrent restoration of predator communities in the upland habitats surrounding the marsh. Thus, both for understanding and enhancing marsh restoration success, we recommend a holistic landscape perspective.

## Supporting information

S1 File Data. Restoration planting data used for this publication.
(XLSX)
